# Acceptance of Medical History-Taking Supported by Artificial Intelligence and Chatbots: A Population-Based Survey in Germany

**DOI:** 10.3390/healthcare14070905

**Published:** 2026-03-31

**Authors:** Sonja Haug, Edda Currle, Karsten Weber

**Affiliations:** Institute for Social Research and Technology Assessment, OTH Regensburg, 93053 Regensburg, Germany; edda.currle@oth-regensburg.de (E.C.); karsten.weber@oth-regensburg.de (K.W.)

**Keywords:** artificial intelligence, history taking, medical, technology acceptance, population survey, UTAUT model

## Abstract

**Highlights:**

**What are the main findings?**

- In a representative survey in Germany with two waves, respondents rated the use of chatbots for medical history positively. Across both survey waves, approval for using a chatbot at home was consistently lower than for using it on site.
- Performance Expectancy, Effort Expectancy and Social Influence explained about half of the variance of behavioral intention in both scenarios. Gender- and age-specific differentiations (except for taking into account the needs of the elderly) are less relevant. Despite the current limited use of the electronic health record (EHR) among patients in Germany, the respondents expressed a high preference for digitally stored medical history data.

**What are the implications of the main findings?**

- Patients are willing to accept learning effort if they are convinced that they will achieve a meaningful performance gain. Simple and intuitive medical history chatbots are needed to ensure accessibility across diverse patient groups.
- AI-supported medical history data has the potential to serve as a pivotal element in the digitalization strategy of medical practices in Germany.

**Abstract:**

**Background/Objectives**: Digital anamnesis tools, including chatbots, are increasingly being developed and evaluated, yet their implementation in German medical practices remains limited. This study examines the acceptance of medical history-taking assisted by artificial intelligence (AI) among the German population. The objective is to derive implications for integrating such systems into digitalization strategies of medical practices. **Methods**: This study is based on an online survey of the German population, aged between 18 and 74 years, conducted in two independent cross-sectional waves (trend design) in 2024 and 2025 with n = 1000 respondents in each year. Based on the Unified Theory of Acceptance and Use of Technology (UTAUT), three hypotheses regarding the use of AI in medical history-taking were developed and tested using linear regression models. **Results**: Both waves reveal a high acceptance level of AI-supported anamnesis systems for people aged between 18 and 74, regardless of whether a chatbot is used in medical practice (Scenario 1) or at home (Scenario 2). The latter received slightly less approval for the intention to use (mean intention scores: 3.50 and 3.45, range from 1.0 to 5.0) than Scenario 1 (3.59, 3.56). The indices of Performance Expectancy (PE), Effort Expectancy (EE), and perceived Social Influence (SI) determine the intention to use a chatbot with the strongest correlation of the PE index (Scenario 1: ß =0.466, Scenario 2: ß = 0.475). Most respondents (73% and 75%) expressed a favorable opinion for digitally storing medical history data within their electronic health record (EHR). **Conclusions**: The findings suggest that gender- and age-specific differentiation—aside from considering the needs of older adults—may be less relevant for designing digitalization strategies than previously assumed. Instead, the focus of medical practices should lie on the practicability of the tool used. Despite currently low EHR utilization rates in Germany, medical practices may expect broad patient approval regarding the digital storage of medical history data.

## 1. Introduction

Medical history-taking is the starting point for further medical treatment. This process involves the documentation of the patients’ present health status and of their medical history, with particular reference to the current symptoms being experienced. Together with the physical examination, it serves as the basis for medical diagnosis. Furthermore, it is conducive to the establishment of a relationship of trust between doctors and patients [1].

Research on the perception of patient safety in outpatient care indicates that the domain of “medical history/diagnostics” is regarded as the medical domain most frequently impacted by safety concerns. The most often perceived problem is insufficient questioning during medical history [2]. In view of the above, digital procedures are being discussed that aim to contribute to patient safety [3,4].

The concept of classic medical history is based on a verbal, personal interview with the patient. Conversely, digital medical history procedures can be conducted without the need for direct personal interaction. The use of technological devices, such as tablets, computers, and smartphones, is a common practice in this context. The implementation of digital medical history procedures is aimed at providing an efficient basis for subsequent consultation and medical diagnosis by doctors. Digital medical history has the potential to reduce the workload of medical professionals. The transfer of the data collection process to patients raises cost-related issues. These considerations apply equally to the preparation of doctor–patient consultations in primary care practices and in specialist practices.

Chatbots in the form of conversational agents (CAs) employ artificial intelligence (AI) to understand questions and provide answers based on Natural Language Processing (NLP). They may be text-based, speech-based, or support both forms. CAs are capable of simulating human conversations. Due to technological progress in NLP, the development of CAs is continuously progressing [5,6]. CAs differ from conventional LLMs or chatbots that can only generate one-time or static responses [7].

AI-based conversational agents (CAs) offer a wide range of applications for healthcare. These include self-management, disease monitoring and health promotion, mental-health counseling and support, addiction therapy, clinical decision-making and diagnostic assistance, optimization of administrative workflows such as appointment scheduling and patient admission, the collection of medical history data, patient education and information, as well as training systems for medical education (patient communication, medical history-taking). The main areas of application are clinical decision support and diagnosis, mental health, and self-management of diseases [6]. Usability issues, complex user interfaces, insufficient integration options with existing systems, and increased computing costs create barriers to acceptance and adoption. Additional challenges include inadequate data protection and data security—particularly when cloud-based systems are used—as well as legal uncertainties regarding liability [7].

The use of chatbots for medical history-taking is currently still largely the subject of research [5]. At present, anamnesis chatbots are regarded as a kind of hypothetical technology that essentially exists as prototypes and—with a few exceptions—has not yet been developed to market readiness.

But it is said that AI has considerable potential for development in the field of digital medical history procedures [8,9]. AI is attributed the capacity to enhance efficiency within the medical history-taking process by utilizing personalized inquiry techniques that are tailored to the specific individual, employing a variety of languages [10] or enabling repeated, automated medical histories. Patients are able to act without the concern of time pressure [11]. The integration of AI into the medical history-taking process has the potential to contribute directly to an increase in the efficiency of healthcare through optimized resource management. Indirectly, it may lead to an enhancement in diagnostic capabilities [9,12].

Medical practices in Germany face increasing pressure to advance their digital transformation. It is widely known that technology acceptance is the key to the actual use of technology. Within the context of medical practices’ digitalization strategies, AI-supported tools for medical history-taking can serve as an essential component. Despite its time-consuming nature, the digitization of this process has yet to be implemented on a wide scale in physician practices with statutory health insurance accreditation [13]. A nuanced understanding of individual behavioral intentions can support healthcare providers in designing and implementing effective digital procedures.

This article was developed as part of a research project analyzing the acceptance of AI-supported applications in medical practices. Its objective is to assess the German population’s acceptance of AI-based medical history-taking procedures, thereby addressing an existing gap in the literature, as no other representative study on acceptance levels in Germany is currently available. The results can be utilized for the selection process of digital technologies within the digitalization strategies of medical practices by including patients’ perspectives into the decision-making process.

## 2. Methods

### 2.1. Study Design

The study group for the study of the acceptance of AI in medical procedures comprises a sample of people from the German population who are over 18 years old. The online survey (computer-assisted web interview) was conducted as a longitudinal study (trend study) with a total of two independent cross-sectional waves from a panel with quotas and weighting. Wave 1 was fielded from 18 to 24 November 2024, and wave 2 from 13 to 17 November 2025. The collection of the data was carried out by NielsenIQ-GfK, Nuremberg, Germany. The survey was part of the online panel GfK eBUS^®^, Nuremberg, Germany. Each sample comprised 1000 individuals aged between 18 and 74, thus representing the German-speaking population. The individuals were not tracked across the waves.

Trend studies are a special form of longitudinal studies. They are used when individual data is not needed and in cases when it is not possible to maintain the connection to the participants. The objective of a trend study is to analyze changes in population over time. This is achieved by conducting surveys of different people in different waves, who nevertheless belong to the same population [14], as, for instance, in [15].

The participants in our study were recruited using a mix of online and offline methods (in person, by telephone and via cooperation partners on the Internet). The quota sampling method is a special form of deliberate selection that aims to match the German population as recorded in the 2022 Micro Census. For the analysis we used a weighting factor created by the survey institute to compensate for any differences between the sample and the population. Non-binary individuals who participated in the study, for example, were randomly assigned to women or men by the survey institute. Only weighted results are reported. Table 1 shows weighted versus unweighted age and gender distribution of both waves. The comparison shows that the sample matches the census.

### 2.2. Theory

**Figure 1:**
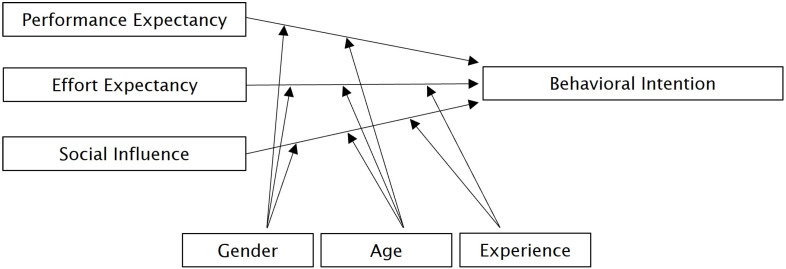
Theoretical model based on the UTAUT model [16].

The theoretical basis for the questionnaire is the “Unified Theory of Acceptance and Use of Technology” (UTAUT) [16]. The UTAUT model and its subsequent versions [17] are widely regarded as a comprehensive theory for understanding technology usage behavior. It is one of the most frequently used models [18,19,20] for analyzing the acceptance and utilization of healthcare technologies and has high explanatory power [21]. For our study the variables were operationalized based on the UTAUT, as originally described in [16] (see Figure 1).

Four constructs influence behavioral intentions to use technology. Firstly, Performance Expectancy (PE) is defined as the extent to which using the technology provides advantages in executing certain activities. In the present study, PE is measured by practicability, treatment quality, and perceptions of data protection and data security. In addition, we considered the aspect of integrating medical history data into an electronic health record (EHR). The following assumptions apply to Performance Expectancy, according to the UTAUT model:

- The higher the perceived practicability of the technology;
- the higher the expectation that the quality of treatment will improve;
- the higher the level of approval for integrating medical history data into the EHR;
  the more likely is the intention to use the technology.

Furthermore, the intention to use the system decreases with perceived risks:

- The higher the fear of misuse of personal data is;
- the higher concerns about data security are;

the more unlikely is the intention to use the technology.

Fear of privacy violations and data misuse can overshadow the functional benefits of a system. Following this assumption, data protection and data security are interpreted as perceived risks that lower expectations that the system will perform as expected. This can have a negative impact on people’s behavioral intentions, making them less inclined to use the system [20,22]. The Likert scales for the perceived risks were reversed for the analysis.

Secondly, Effort Expectancy (EE) refers to the perceived difficulty of using a system. The easier people perceive technology is to use, and the more confident they are in their ability to use it, the greater their intention to use it. Thirdly, Social Influence (SI) reflects respondents’ subjective expectations of how medical staff would judge their use of technology. If the medical staff welcome the implementation of new technology, this appears to have a positive effect on the intention of patients to use it.

The impact of these three constructs on behavioral intention is influenced by individual differences in the variables gender, age, and experience, which act as moderator variables. According to the UTAUT model gender is used as a social construct, explaining differences that may arise from gender roles and socialization [16], and may contribute to inequalities in access to healthcare [23].

### 2.3. Generation of Hypothesis

Three central hypotheses were generated from the UTAUT model. For calculations in this paper, data collected in the second wave was used (see also: [24]).

**Hypothesis** **1** **(H1):** *Higher expectations of the technology’s performance are associated with a stronger behavioral intention to use it [19,20,25]. The influence of Performance Expectancy on behavioral intention is moderated by gender and age. The effect is expected to be more pronounced among men, especially younger men [16,19].*

**Hypothesis** **2** **(H2):** *The lower the expected effort required to use the system is, the higher the behavioral intention [19,20,25]. The influence of Effort Expectancy on behavioral intention is moderated by gender, age, and previous experience with digital recording of patient and health data in the anamnesis process. It is assumed that the Effort Expectancy is more pronounced in women, particularly in the elderly with no experience of digital recording of patient and health data [16,19]*.

**Hypothesis** **3** **(H3):** *The higher the perception of Social Influence, i.e., the higher the patients’ perceptions that the medical would appreciate their use of the technology, the higher the intention to use the system is [19,20,25]. Social Influence is moderated by gender, age, and experience with digital recording of patient and health data in the anamnesis process. It is assumed that women and the elderly perceive Social Influence more strongly when it comes to the intention to use a new technology, with the effect decreasing with existing experience [16,19].*

### 2.4. Survey Instrument

The questionnaire, which was developed based on the UTAUT model, consists of nine standardized questions [26]. The questions operationalize the model’s constructs. Items surveying experience, as well as aspects of data protection, data security, and integration into EHR, were developed in-house. To analyze the dependent variable of usage intention, two scenarios representing different usage contexts were designed. Scenario 1 involves using a chatbot in a doctor’s office, while Scenario 2 describes using a chatbot at home prior to a doctor’s appointment.

Other items are based on existing studies on the acceptance of technology in different fields of application. These were partially translated and adapted for the specific context of this study. The item concerning personal assessment when dealing with an AI-supported questionnaire was taken from Venkatesh et al. [16] The item concerning the effort to use a chatbot was taken from a study by Heerink et al. on the acceptance of social robotics [27]. The item on Social Influence was also taken from this source. The practicability question is based on the item concerning Perceived Usefulness from the same study and corresponds to the UTAUT construct of Performance Expectancy. The item addressing expectations regarding the quality of medical treatment is based on the acceptance study of Arora et al., in which an automated system for collecting medical histories in emergency rooms was evaluated [28].

Furthermore, the item on Technology Affinity is drawn from the concise scale for evaluating technology readiness, as outlined by Neyer et al. [29], while the question on Technology Competence was derived from a study conducted by Seifert et al. on the utilization of information and communication technology by older adults [30]. The item on Technology Use was developed based on a study evaluating technology for promoting the mobility of the elderly [31]. The original question was supplemented with additional devices. The questions concerning socio-demographic characteristics and trend types were formulated by the commissioned survey institute.

Attitudes were measured using a five-point Likert scale (1 “Strongly disagree”, 2 “Disagree”, 3 “Neither agree nor disagree”, 4 “Agree” and 5 “Strongly agree”). Five-step scales were used in the same way as metric scales [32]. The options “I don’t know” and “No response” were excluded from the analysis, resulting in varying numbers of valid cases. The questionnaire [26] was pretested, including a test to measure cognitive ability with three subjects. The survey took an average of seven minutes to complete.

### 2.5. Evaluation Strategy and Statistical Analyses

The demographic factors of age and gender of the two samples, which were essential for the analysis in the model, were processed using a univariate analysis. A univariate analysis of the technology acceptance aspects of the samples was also carried out. In addition, the independent variable of experience and the dependent variable of intention to use were processed descriptively for both data sets and for both scenarios.

To form indices based on the independent variables that belong to the same UTAUT construct, the reliability of the items was calculated. VIF values were calculated to identify potential multicollinearity. Tests for normal distribution were conducted by examining residual plots and using Kolmogorov–Smirnov tests. Tests for heteroscedasticity were performed using Breusch–Pagan tests.

The hypotheses derived from the model have previously been tested for the first wave [14]. As the use of the EHR has only been required for hospitals, pharmacies, and doctors’ surgeries in Germany since October 2025, this item was not included in the analysis of the first wave. For the paper at hand, bivariate correlation analyses and multiple linear regression analyses to determine the relationships between the independent and dependent variables and to test the hypotheses of the model were performed on the data from the second wave (2025). The EHR item has now been included. The analyses were calculated using IBM Statistics SPSS 29.

## 3. Results

The sample is described below. Selected descriptive results are followed by testing the hypotheses for the second wave of the survey.

### 3.1. Sample

The samples from both survey waves (wave 1: n = 1000, wave 2: n = 1001) represent the German-speaking population aged 18–74 (the average age in both waves is 47.2 years, median age 2024: 47.8; 2025: 48.0; SD 2024: 15.5; 2025: 15.8). Each sample was composed of 50% women and 50% men. Around one-fifth of respondents had a low school-leaving qualification and just under one-third had an intermediate one. Around 21% had a university entrance qualification, while a further 25% stated that they had acquired an academic degree.

### 3.2. Aspects of Technology Acceptance

The results for the selected aspects of technology acceptance are essentially consistent across both samples.

Technical Competence: In the first and second waves, one-fifth of respondents “strongly agreed” or “somewhat agreed” with the statement that they find it difficult to operate modern technical devices. By contrast, just under two-thirds were confident in their ability to use modern technology, agreeing “somewhat” or “not at all” with the statement. A further 16% were undecided on this question.

Technological Affinity: In both waves, more than 60% of respondents stated that they quickly take a liking to new technological developments. Around 16% “somewhat disagreed” or “strongly disagreed” with this statement. Around one-fifth of respondents were undecided.

Trend Types: The sample from the second wave can be considered slightly more conservative in terms of Trend Types. While one third of respondents in the first wave stated that they were very interested in new trends or developments (“innovative”), this proportion fell to around 30% in the second wave. More than half of respondents would prefer to wait and see whether a trend proves itself (“wait-and-see”: 53% in 2024; 51% in 2025). Around 13% of respondents to the first survey prefered to stick with the tried and tested (“conservative”). This proportion increased to 18% in the second wave. However, the Trend Types do not refer explicitly to technological developments but also include developments in other areas.

Technology Use: Around 14% of respondents regularly used only one device, or none, from a selection of five types (computer/laptop, smartphone, tablet, fitness wristband/smartwatch, or voice assistant), in their everyday private life. Around 60% and 62% of respondents, respectively, used two or three devices regularly. In the first survey, more than a quarter of participants stated that they regularly used four or all the listed devices. This proportion fell to around 23% in the second wave of the survey.

### 3.3. Experience

Compared to the first survey, experience with digital medical histories increased slightly, while experience with chatbots remained constant. Examining the survey data reveals that in 2024 almost two-thirds (63%) of respondents still had no experience with digital medical history. They answered “no” to the question of whether they had ever entered their medical history data themselves on a computer, tablet, or smartphone when visiting a doctor’s office. Just under 4% were unsure. The remaining third were asked whether artificial intelligence, e.g., in the form of a chatbot, had been used in the digital recording they mentioned. Just under a quarter of respondents agreed with this statement, while around 59% disagreed. The proportion of respondents who expressed a degree of uncertainty about this question was significantly higher than for the question about digital recording. Wave 2, which was conducted the following year, showed higher experience. Only 57% of respondents stated that they had no experience with digital medical history-taking. In contrast, the proportion of experienced individuals rose to around 39%. The proportion of participants who stated that they had experience of using chatbots remained stable (at around one-fifth), as did the degree of uncertainty around this question.

### 3.4. Comparison of Two Scenarios for the Use of AI-Supported Procedures in the Anamnesis Process

#### 3.4.1. Behavioral Intention

**Figure 2:**
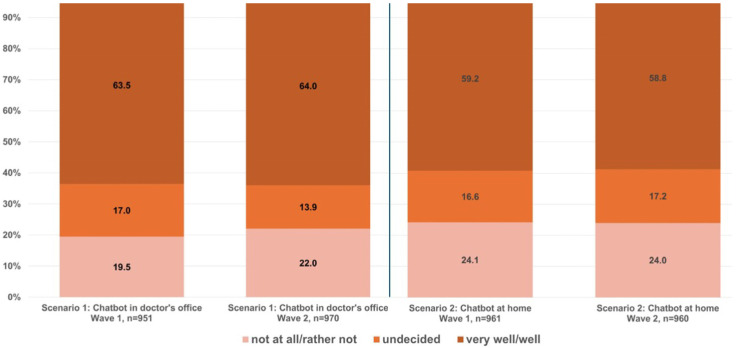
Intention to use a chatbot for anamnesis in two scenarios (wave 1 and 2).

The findings of both waves indicate a high acceptance level of AI-supported anamnesis systems for people aged between 18 and 74. In the first wave, approximately two-thirds of respondents said they could “very well” or “well” imagine using a chatbot to take their medical history in a doctor’s office. Around 17% were undecided. A mere fifth stated they could “somewhat” or “not at all” imagine this (mean value 3.59, range from 1.0 to 5.0). Scenario 2, involving taking medical histories with a chatbot at home prior to the doctor’s appointment, received slightly less approval. Almost a quarter of the sample could “somewhat” or “not at all” envisage this scenario, whereas the rate for (strong) agreement was 59.2% (mean value 3.50) (see Table 2 and Figure 2).

The approval ratings for the two scenarios remained stable in the second survey wave. Around 64% of respondents could “very well” or “well” imagine using a chatbot in a doctor’s office (average value 3.56). As in wave 1, Scenario 2 received slightly less approval than Scenario 1, at 59%. Almost a quarter of participants expressed a preference for avoiding or rejecting Scenario 2 in 2025 with an average value of 3.45 (see Table 2 and Figure 2).

#### 3.4.2. Performance Expectancy, Effort Expectancy and Expectations Regarding Social Influence

Table 3 shows a comparison of the mean values of the UTAUT model’s independent variables. Low agreement values for a construct indicate that it poses a risk to the intention to use technology (mean below 3). Conversely, values above 3 represent an opportunity for behavioral intention.

The concerns about data protection (mean value 2.69) and data security (mean value 2.62) are likely to prevent the participants of wave 2 from using AI for medical history-taking. The potential improvement in the quality of medical treatment using AI in medical history-taking was identified neither as an opportunity nor as an explicit risk (with a mean value of 3.19). In contrast, both practicability (3.69) and possibility to store data in the EHR (3.98) are seen as an opportunity. Furthermore, the sample is on average positive about the expected effort involved in using the system. Overall, the values for the standard deviations, which are consistently greater than the value of 1 for all variables, suggest a high level of dispersion within the sample.

The Performance and Effort Expectancy constructs were combined to form an additive index. Cronbach’s α was used to assess the reliability of the PE and EE items to form an index. The value determined for the PE index is 0.733. The Cronbach’s α value for the EE constructs was 0.696 (wave 2). Consequently, both constructs are reliably represented in the model. PE and EE indices were computed as the mean of their items (range: 1 = low to 5 = high). Higher values indicate more favorable expectations. Both the response “I don’t know” and missing values were excluded from the computation of the indices. Cronbach’s alpha values were calculated in both waves and remained stable, thus continuing to perform well.

Table 4 shows that most respondents (n = 888) feel confident in their ability to use digitally supported medical history-taking procedures (measured by mean EE index with a total mean value of 3.80). A descriptive analysis shows that this effect decreases with age. Older people are more likely than younger people to assume that learning to use technology will require more effort. Comparing the mean PE index values (n = 838) across age groups shows only insignificant differences. Whereas concerns about data protection remain below the neutral midpoint (see Table 3), the total mean value of the PE-Index is moderately positive (3.26). The mean value of SI indicates the most positive score, 3.84 (n = 899). In this context, the oldest respondent group demonstrated the lowest SI construct value, 3.33, indicating a lesser degree of influence of opinions of others compared to younger groups.

#### 3.4.3. Influence of Performance Expectancy, Effort Expectancy and Social Influence on the Intention to Use

In the bivariate analysis, all the constructs expected to influence behavioral intention showed significant correlations to it. This applied to both scenarios. Practicability was found to be the factor most strongly associated with Scenario 1 and Scenario 2. The summarized index for Performance Expectancy showed a strong positive correlation with both scenarios, while the index for Effort Expectancy showed a moderate positive correlation. The correlation coefficient for expected Social Influence was slightly higher for use at the doctor’s office than for Scenario 2 with use at home. None of the bivariate correlations between the independent variables exceeded the value of 0.620 (for data protection and data security).

A multiple linear regression model using Scenario 1 (use in doctor’s office) as the dependent variable shows significant effects for all items except data protection and the expected effort for the use of a KI-supported questionnaire, albeit weak ones. The multicollinearity check for Scenario 1 did not reveal any critical VIF values, with the highest VIF value recorded at 1.89 and a tolerance value of 0.53 (Practicability), indicating that there is no significant multicollinearity among the predictors. The robustness checks with the modified Breusch–Pagan test revealed heteroscedasticity. Consequently, heteroscedasticity-consistent standard errors are additionally documented for Scenario 1. Practicability proved to be the item with the strongest effect. In other words, the higher the expectation that the system would be practical, the higher the acceptance of using a chatbot in the doctor’s office. The model showed moderate goodness of fit (R^2^ = 0.514) (Table 5). The answer options “I don’t know” and “No response” were excluded from the analysis.

Identical effects were observed in a second model with the independent variable in Scenario 2 (use of AI at home before visiting a doctor’s office). Once again, there is no significant multicollinearity among the predictors while the robustness checks with the modified Breusch–Pagan test revealed heteroscedasticity. Heteroscedasticity-consistent standard errors are documented in addition. In the context of Scenario 2, a weakly positive significant impact of Practicability was identified, but no effect of data protection or Expected Effort for AI-supported questionnaires. By contrast, the impact of Expected Social Influence proved to be of a lesser degree in Scenario 2. The model demonstrated a moderate degree of goodness of fit, indicated by R^2^ = 0.5. In both models, the data integration into the EHR variable showed statistically significant correlations. The response options “I don’t know” and “No response” were excluded in the regression analysis (see Table 6).

Further analysis examined the effects of the indices formed from individual variables. Multicollinearity checks for both scenarios did not reveal multicollinearity among the indices as predictors, whereas the robustness checks with the modified Breusch–Pagan test revealed heteroscedasticity. Heteroscedasticity-consistent standard errors are consequently documented for each model. In summary, the UTAUT constructs, i.e., the summarized indices and the one-item Social Influence construct, showed the expected positive effects on the intention to use either a chatbot at home or at the doctor’s office. The Performance Expectancy index exhibited the strongest correlation in each scenario. Both models demonstrate moderate goodness of fit (see Table 7).

### 3.5. Integration of Medical History Data into Electronic Health Records (EHRs) Before and After 2025

Following the nationwide implementation of the electronic health record (EHR) in Germany in 2021, its adoption remained minimal, with less than 1% of individuals covered by statutory health insurance using the system as of July 2022. Statutory health insurance in Germany is mandatory and funded through insurance premiums. The limited uptake of the EHR is primarily attributed to insufficient public awareness and the restricted availability of the EHR in medical practices. Moreover, in 2022 the system was still based on an opt-in approach, requiring approval and active registration by users. A population-based survey conducted in July 2022 confirmed the low level of awareness among insured individuals. Approximately one-third of respondents indicated an intention to use the EHR in the future [33].

The German Digital Act, which is short for the Act on Accelerating Digitalization in Healthcare, established an opt-out principle for the EHR. It came into effect on 15 January 2025. Since then, people with statutory health insurance have had an EHR created for them automatically, unless they actively object and opt-out. They also decide which data is allowed to be stored or deleted. The data that might be stored in the EHR includes, among other information, general information about the patient and diagnoses. Data collected as part of a medical history is not yet explicitly stored. The medical history sheet is set to be integrated later [34]. Since 1 October 2025, the use of the EHR is required for hospitals, pharmacies, and doctors’ surgeries in Germany.

**Figure 3:**
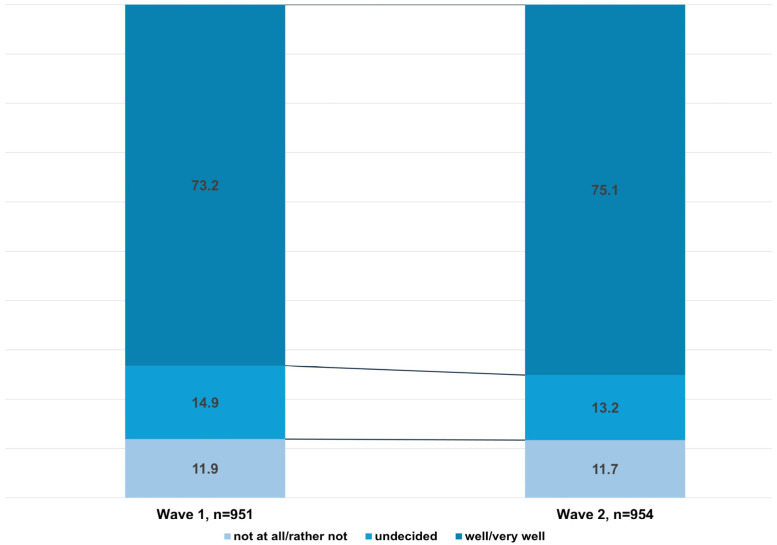
Attitudes towards storing medical history data in electronic health records (wave 1 and 2).

Even before the nationwide introduction of electronic health records, around 73% of participants in wave 1 were in favor of storing their medical history collected via chatbots in their patient records. In wave 2, this proportion slightly rose to 75%. The proportion of those who rejected this storage remained constant (see Figure 3). A statistically significant, moderate positive correlation was found between the intention to use a chatbot and the integration of data into the EHR, in both waves and for both scenarios.

The acceptance rates for the idea of storing anamnesis data in the EHR demonstrates a high contrast to its adoption rates. Despite the obligation of healthcare providers in Germany to fill in the EHR, introduced in October 2025, both the number of patients registered for use and the number of people actively using the EHR fell short of expectations. The EHR therefore still cannot be regarded as a central component of digitalization in healthcare [35]. This might hinder the adoption of other digital services like chatbots.

### 3.6. Results of Hypothesis Testing

A further regression analysis was carried out to test the hypotheses and examine how the moderator variables gender, age, and experience affect the findings. This reveals which variables have a statistically significant moderating effect on the PE and EE indices, as well as on the SI item (see Table 8).

The results of the linear regression analyses show that the hypotheses derived from the UTAUT model could only be partially verified. Hypothesis 1 was confirmed, as expectations regarding the performance of a system determined behavioral intention, meaning that the higher the expectations, the more likely respondents were willing to use the technology. There were no gender differences and no statistically significant correlations between PE and intention to use, moderated by age, could be established either. The assumption that the effect would be more pronounced among younger people was therefore not confirmed.

Hypothesis 2 was also confirmed, showing that expectations regarding the effort required to use a system influence behavioral intention. The lower the Expected Effort, the more likely respondents were to say they could imagine using the technology. This was shown for the data from wave 2 by both the bivariate correlations and the results of the regression analyses. However, neither age and gender nor previous experience had a statistically significant effect on the correlation between EE and intention to use.

The perception of Social Influence was also related to the behavioral intentions of the respondents (Hypothesis 3). This applied to both usage scenarios. The bivariate analysis showed that older respondents tended to be more “immune” to social expectations. This was confirmed by the moderation regression analysis where reverse age effects were observed for both scenarios. Contrary to the UTAUT hypothesis, younger age groups were more likely to perceive Social Influence. Experience, on the other hand, did not prove to be a moderator variable for Social Influence.

## 4. Discussion

### 4.1. Aim and Contribution of This Study

There is currently no other data available on patient attitudes towards the introduction of AI in the medical history-taking process in German medical practices. The present study addresses this research gap by presenting results on the acceptance of AI-supported applications in the medical history-taking process from patients’ perspective. The findings indicate a high overall intention to use for both scenarios for people aged between 18 and 74.

The present findings are consistent with the results of a comparative study on digital use cases for medical practices. It was demonstrated that patients particularly appreciate the digital provision of information prior to the medical consultation [36]. Our results show that the use of a chatbot in the domestic environment has received lower approval ratings across both waves in comparison to on-site use. The observed difference can be attributed to two factors. First, the requirement to use personal devices, and second, the absence of opportunity for direct consultation with medical professionals during the data collection process [37]. A patient survey noted that some respondents generally preferred personal consultation [38].

In view of our results, medical offices can draw the conclusion that most of their adult patients under the age of 75 are not opposed to the use of AI in the medical history-taking process. Usability studies on mobile AI-based applications conducted in German-speaking countries [39,40] have even pointed out that chatbots motivate patients to provide more thorough answers to medical history questions. The data collected in Germany can be used as a reference point for countries with similar healthcare systems and population structures. We believe that further research is needed in order to support medical practices in other countries within their digitalization strategies by including patients’ perspectives into the decision-making process.

### 4.2. Discussion of the Results in the Context of Existing Research

Although the development of digital medical history applications can be traced back to the mid-20th century [8], the development of technical solutions for digital medical history-taking has focused primarily on the clinical field. A meta-study published in 2022 concluded that only 17% of the systems examined used AI [41]. Furthermore, a limited number of studies considered patient acceptance [41,42].

Since 2020, there has been a significant increase in studies on the use of digital methods in the medical history-taking process and its consequences [9,42]. But it is evident that aspects of acceptance were inadequately addressed. Conversely, the emphasis has been predominantly on the efficiency and quality of care (usefulness) or on the usability of prototypes [42,43]. Usability studies generally focus on specific patient groups [39,44,45] or areas of application [5,38,46,47]. Only a small number of prototypes have been developed and evaluated for primary medical care. Notable exceptions are the studies by Ni et al. [48] or Verma et al. [49]. The latter examined the use of a chatbot for telemedical care in India. Another feasibility and acceptance study analyzed the use of an AI chatbot for comprehensive medical history-taking in outpatient primary care based on a small number of cases [50].

The central assumptions of the UTAUT model regarding the factors influencing the intention to use were confirmed by the results. In line with the results of other studies on technology acceptance in the healthcare sector, it can be concluded that the UTAUT constructs have a significant influence on the acceptance of digital (medical history-taking) procedures [36]. A review on the acceptance of digital health technologies with a particular focus on conversational agents (CAs) in various domains has confirmed the validity of the UTAUT model [18]. This finding is supported by studies on the acceptance of AI health assistants in China [20,51].

The present study confirms that PE is the strongest predictor of behavioral intention, as has been demonstrated in previous research [19,36,50]. Hong et al. [50] demonstrated that the majority of patients believe that AI-based systems could help doctors to adequately understand their health status and assist them in identifying health risks. This finding is consistent with the results of our study on respondents’ expectations regarding improvements in the quality of treatment. As a decision-making aid and takeaway for medical practices, it can be noted that patients are willing to accept learning effort if they are convinced that they will achieve a relevant gain in performance.

Other studies have identified concerns regarding data protection and data security as significant barriers to the use of chatbots in healthcare [21]. In contrast to the results of the first wave [24], data protection was not statistically significant in the regression analysis for either of the two scenarios in wave 2 of the survey. Although the results of the second wave indicate that the intention to use chatbots is not significantly reduced among respondents who perceive risks in relation to data protection, the collection and storage of health data nevertheless place special demands on both data protection and data security for medical practices.

In addition to the aspects mentioned previously, practicability is of crucial importance for the implementation of AI-supported systems in medical practices. The analysis demonstrated that practicability of a system has the highest explanatory power. Respondents who attached great importance to practicability were more inclined to use a chatbot, irrespective of whether they were at home or present at a medical facility. This results in the requirement to design medical history-taking chatbots for medical offices that are simple and intuitive for all user groups, regardless of the age of the patients.

The findings of the moderator analysis indicate that the moderating variables exerted only a limited effect and behave differently than UTAUT originally proposed. The effects expected for the moderating factor of gender could not be demonstrated in the present study for the age group 18–74. This finding contradicts the assumptions of the UTAUT model, which postulates gender-specific differences regarding the influencing factors. Other studies based on the UTAUT model also indicated this finding of limited effects of gender [19,52].

Contrary to the prevailing hypothesis concerning the moderation of Social Influence by age, our findings demonstrated a reverse age effect. The hypothesis that older people attach greater importance to the opinions of others when using new technology was therefore not supported by the results [19].

In both scenarios, evidence was found for age-dependent influences on performance expectations and on usage intention, albeit only weak effects. This may be attributed to the increasing adoption of digital technologies and applications, such as smartphones, health-related apps, and websites among the German population over the age of 50 [53]. In mature digital societies, socio-demographics may be less informative than perceived value and usability for predicting health technology use. However, it should be noted that the sample in our survey did not include people over the age of 75. In this context, it is noteworthy that the proportion of “off-liners” in Germany among 76–90-year-olds remains high at approximately 40%, despite a decline. A slight majority of respondents within this age group report frequent use of the Internet for information purposes [54].

### 4.3. Findings in Relation to Current Challenges in Germany

It can be assumed that age (over 75) can still act as a potential barrier to the use of AI-supported technologies in healthcare. This barrier can be attributed to the limited digital skills of very old people [18]. Simultaneously, individuals over the age of 75 are particularly affected by the introduction of new technologies such as chatbots due to their increased need for care. Due to age-related limitations, this group is particularly dependent on smooth healthcare provision and barrier-free access to (digital) healthcare services. In consideration of the probable increase in the significance of self-management in healthcare, it is vital to methodically consider the particular requirements of the very old and disabled when designing digital applications, both in the present and in the future [18]. This is of paramount importance for strategies for implementation of digital technologies in medical practices. In view of the aforementioned context, it is evident that more research on acceptance and utilization of digital technologies within the German healthcare system is required, with a particular emphasis on the perspective of elderly individuals [41].

The majority of respondents expressed support for the storage of medical history data in the EHR. However, the extent to which these respondents utilize their EHR remains uncertain. Despite the nationwide roll-out, active use of the EHR in Germany is still developing slowly. Although abstract approval ratings for the EHR have risen over time [33,55], few of those with statutory health insurance actively use it [56]. The complexity of the setup and the absence of user-friendliness hinder active use.

The results of our research demonstrate that most respondents expressed not only a high level of acceptance for the digital collection of medical history data, but also largely approved storage of this data in the EHR. Medical practices opting for AI-supported collection of medical history data can therefore anticipate that the integration of this data into electronic patient records will be accepted by most of their patients. This underscores the need to continue pursuing the process of promoting active access to EHR by addressing the secondary factors (level of information, habits, and data protection) that affect first-time use [35].

### 4.4. Limitations

The present study has limitations that must be considered when interpreting the results. A notable limitation of this study is the exclusion of individuals over the age of 75, as this age group is not represented in the panel. We recommend conducting additional research on acceptance among the very old.

AI implementation, particularly in the form of chatbots, has yet to become widespread in the German healthcare system. Regarding technologies that are not yet widespread, acceptance research faces the challenge of surveying hypothetical intentions to use them. Furthermore, it cannot be ruled out that questions regarding chatbots were met with incomprehension by some sections of the population. This is evidenced by the finding that approximately 20% of respondents expressed uncertainty when asked whether AI had ever been utilized in their medical history-taking process.

A selection bias cannot be ruled out, as participants in an online panel may be more tech-savvy, tech-competent and innovation-friendly than a cross-section of the population based on a random sample. In comparison to the cross-section of patients in medical practices, the present sample includes a higher proportion of younger and more highly educated individuals, with the distribution approximating that of the general population. To address this selection bias, we recommend further research using CATI (Computer Assisted Telephone Interview).

As the data has been collected in Germany, it cannot be readily applied to other countries. Instead, it can serve as a point of reference for countries with similar healthcare systems and demographic structures, which, we believe, indicates a need for further research. This might support medical practices in other countries in developing their digitalization strategies by including patients’ perspectives into the decision-making process.

## 5. Conclusions

The results of this study are relevant in the context of the pressure to digitize medical practices in Germany. A differentiated understanding of individual behavioral intentions can assist in the development and implementation of AI-supported medical history-taking procedures. While gender- and age-specific differentiations (except for considering the needs of the elderly) may be less relevant than previously assumed in the design of digitization strategies, the focus should be on the practicability of the tools, applications, and devices used. When introducing AI-supported anamnesis, medical practices should first ensure that tools are easy to operate and clearly improve perceived efficiency, as these factors strongly shape patient acceptance.

Furthermore, the importance of engaging diverse interest groups has been demonstrated [42]. The present study indicates that the perceived approval of practice staff for the utilization of digital applications exerts a positive influence on patients’ intention to employ them. Therefore, it is crucial to involve practicing staff in implementation processes at an early stage in order to ensure their acceptance and thus the sustainable use of digital medical history-taking tools [18,42]. Digitalization strategies should move beyond age- and gender-related assumptions and instead focus on cross-cutting usability, communication, and data protection concerns.

Despite the EHR’s current utilization by only a limited number of patients in Germany, medical offices can presume a general patient preference for the digital storage of medical history data within the EHR.

In light of the rapid increase in scholarly publications concerning the use of chatbots in healthcare [57] and the automation of medical history-taking [9,41], recent research has sought to address existing gaps in the field. The demand for a standardized procedure for evaluating the usability of chatbots [58] was taken up presenting a uniform evaluation scheme for evaluating the conversational reasoning capabilities of clinical LLMs [4].

Future interactions between patients and medical staff will increasingly take place mediated by technology, particularly by AI in medical history-taking. Both sides of the dyadic system consisting of doctors and patients are affected by these changes. However, the investigation of ethical, legal, and social aspects of this change in interaction—i.e., as documented in [8,59]—is a research desideratum that continuously must be taken into account and in parallel with technological progress [9]. It is only under this premise that “a tenable balance between the use of AI-based technologies and maintaining a human and accountable relationship between medical professionals and their patients” [59] can be achieved. Furthermore, research on inequalities arising from the utilization of AI in healthcare is essential [60].

## Figures and Tables

**Table 1:** Comparison of weighted and unweighted age and gender distribution in both waves.

	Weighted	Wave 1 Unweighted	Wave 2 Unweighted
Men	50.1%	50.3%	48.4%
Women	49.9%	49.7%	51.6%
Average age	47.2	48.4	49.1

**Table 2:** Behavioral intention for both waves and scenarios: core values and rates for (strong) agreement.

	Wave 1		Wave 2	
	Scenario 1	Scenario 2	Scenario 1	Scenario 2
Mean	3.59	3.50	3.56	3.45
SD	1.19	1.24	1.27	1.31
Proportion of (strong) agreement	63.5%	59.2%	64.0%	58.8%
n	951	961	970	960

**Table 3:** Mean values for the independent variables of PE, EE, and SI (wave 2).

	Construct	Mean Values	SD	n
Attitudes				
Data protection	PE	2.69	1.27	930
Data security	PE	2.62	1.26	970
Practicability	PE	3.69	1.14	955
Quality of treatment	PE	3.19	1.22	935
Integration of data into EHR	PE	3.98	1.15	954
Expected effort for chatbot	EE	3.94	1.10	939
Expected effort for AI-supported questionnaire	EE	3.65	1.25	921
Expected Social influence	SI	3.84	1.08	899

PE: Performance Expectancy; EE: Effort Expectancy; SI: Social Influence; five-point scale from 1: strongly disagree to 5: strongly agree.

**Table 4:** Mean values for the indices of PE, EE, and SI by age and gender (wave 2).

Age Group	Gender	Mean Value PE Index	Mean Value EE Index	Mean Value SI
18–29	Male	3.28	4.09	3.83
	Female	3.35	4.02	3.98
	Total	3.32	4.05	3.91
30–49	Male	3.38	4.07	4.07
	Female	3.22	3.84	4.04
	Total	3.30	3.96	4.06
50–69	Male	3.25	3.76	3.71
	Female	3.18	3.49	3.67
	Total	3.22	3.62	3.69
over 70	Male	3.00	3.26	3.24
	Female	3.18	3.29	3.42
	Total	3.10	3.28	3.33
Total	Male	3.28	3.91	3.83
	Female	3.23	3.69	3.84
	Total	3.26	3.80	3.84

PE: Performance Expectancy; EE: Effort Expectancy; SI: Social Influence; Additive index: 1: low, 5: high.

**Table 5:** Determinants of behavioral intention in Scenario 1: Use of a chatbot in doctor’s office (linear regression, wave 2).

	Item	Scenario 1 Use in Doctor’s Office					
		ß	HCSE	95% CI Lower Limit	95% CI Upper Limit	VIF	Tolerance Value
PE	Data protection	0.023	0.040	−0.040	0.115	1.757	0.569
	Data security	0.197 ***	0.040	0.101	0.259	1.847	0.541
	Practicability	0.262 ***	0.048	0.213	0.401	1.886	0.530
	Quality of treatment	0.089 *	0.035	0.015	0.153	1.530	0.654
	Integration of data in EHR	0.178 ***	0.037	0.110	0.257	1.480	0.675
EE	Effort for chatbot	0.148 ***	0.044	0.085	0.258	1.591	0.629
	Effort for AI-supported questionnaire	0.065	0.035	−0.006	0.132	1.559	0.642
SI	Medical staff	0.126 ***	0.036	0.081	0.223	1.311	0.763
n		738					
R^2^		0.514					

Dependent variables: intention to use chatbot in doctor’s office; five-point scale from 1: can’t imagine at all to 5: can imagine very well; * *p*-value < 0.05; *** *p*-value ≤ 0.001; ß = standard beta coefficient; HCSE = heteroscedasticity-consistent standard errors, HC3; CI = confidence interval; VIF = variance inflation factor; R^2^ = coefficient of determination.

**Table 6:** Determinants of behavioral intention in Scenario 2: Use of a chatbot at home (linear regression, wave 2).

	Item	Scenario 2 Use at Home					
		ß	HCSE	95% CI Lower Limit	95% CI Upper Limit	VIF	Tolerance Value
PE	Data protection	−0.010	0.042	−0.074	0.090	1.754	0.570
	Data security	0.199 ***	0.041	0.103	0.265	1.835	0.545
	Practicability	0.265 ***	0.047	0.247	0.434	1.875	0.533
	Quality of treatment	0.167 ***	0.036	0.102	0.243	1.523	0.657
	Integration of data in EHR	0.150 ***	0.038	0.090	0.237	1.490	0.671
EE	Effort for chatbot	0.145 ***	0.047	0.074	0.257	1.590	0.629
	Effort for AI-supported questionnaire	0.067	0.034	−0.006	0.127	1.543	0.648
SI	Medical staff	0.064 *	0.035	0.011	0.150	1.306	0.765
n		736					
R^2^		0.500					

Dependent variables: intention to use chatbot in doctor’s office; intention to use chatbot at home; five-point scale from 1: can’t imagine at all to 5: can imagine very well; * *p*-value < 0.05; *** *p*-value ≤ 0.001; ß = standard beta coefficient; HCSE = heteroscedasticity-consistent standard errors, HC3; CI = confidence interval; VIF = variance inflation factor; R^2^ = coefficient of determination.

**Table 7:** Influence of indices on behavioral intention in two scenarios (linear regression, wave 2).

Independent Variables	ß	HCSE	95% CI Lower Limit	95% CI Upper Limit	VIF	Tolerance Value
	Scenario 1 Use in doctor’s office					
Index Performance Expectancy	0.466 ***	0.040	0.583	0.740	1.141	
Index Effort Expectancy	0.252 ***	0.038	0.224	0.372	1.331	
Expected Social Influence	0.156 ***	0.037	0.112	0.258	1.256	
n	738					
R^2^	0.454					
	Scenario 2 Use at home					
Index Performance Expectancy	0.475 ***	0.043	0.632	0.799	1.140	0.876
Index Effort Expectancy	0.265 ***	0.041	0.234	0.397	1.323	0.751
Expected Social Influence	0.101 ***	0.037	0.053	0.199	1.251	0.796
n	736					
R^2^	0.436					

Dependent variables: intention to use chatbot in doctor’s office; intention to use chatbot at home; five-point scale from 1: can’t imagine at all to 5: can imagine very well; *** *p*-value ≤ 0.001; ß = standard beta coefficient; HCSE = heteroscedasticity-consistent standard errors, HC3; CI = confidence interval; VIF = variance inflation factor; R^2^ = coefficient of determination.

**Table 8:** Moderation of age, gender, and experience on PE, EE and SI in two usage scenarios (linear regression, wave 2).

	Scenario 1 Use in Doctor’s Office	Scenario 2 Use at Home
	ß	ß
Moderator variable Age/PE	0.070 **	0.068 *
Moderator variable Gender/PE	−0.036	−0.014
Moderator variable Age/EE	0.029	−0.006
Moderator variable Gender/EE	−0.043	−0.033
Moderator variable Experience/EE	0.050	0.020
Moderator variable Age/SI	−0.073 *	−0.112 ***
Moderator variable Gender/SI	0.068	0.039
Moderator variable Experience/SI	−0.004	−0.004
R^2^	0.466	0.461

PE: Performance Expectancy; EE: Effort Expectancy; SI: Social Influence; dependent variables: intention to use chatbot in doctor’s office; intention to use chatbot at home; five-point scale from 1: can’t imagine at all to 5: can imagine very well; * *p*-value < 0.05; ** *p*-value ≤ 0.01; *** *p*-value ≤ 0.001; ß = standard beta coefficient; R^2^ = coefficient of determination.

## Data Availability

The original data “Künstliche Intelligenz und Anamnese (KI-Anamnese)—Datensätze Welle 1 und Welle 2” presented in this study is publicly accessible in Zenodo at DOI 10.5281/zenodo.18017447. The files can be accessed by users upon request from the corresponding author.

## Abbreviations

The following abbreviations are used in this manuscript:

AI	Artificial Intelligence
CA	Conversational Agent
CATI	Computer Assisted Telephone Interview
EE	Effort Expectancy
EHR	Electronic Health Record
LLM	Large Language Model
NLP	Natural Language Processing
PE	Performance Expectancy
SI	Social Influence
UTAUT	Unified Theory of Acceptance and Use of Technology
